# Flow visualization data from experiments with an oscillating circular cylinder in a gravity-driven soap film

**DOI:** 10.1016/j.dib.2022.107819

**Published:** 2022-01-19

**Authors:** Emad Masroor, Wenchao Yang, Mark A. Stremler

**Affiliations:** a Engineering Mechanics Program, Virginia Tech, Blacksburg VA, 24061, USA; b Department of Mechanical and Materials Engineering, Queens University, Kingston, Ontario, K7L 3N6, Canada; c Department of Biomedical Engineering and Mechanics, Virginia Tech, Blacksburg, VA, 24061, USA

**Keywords:** Vortex-induced vibrations, Vortex dynamics, Soap films

## Abstract

This article contains flow visualization data from experiments conducted in an inclined gravity-driven soap film intersected by a circular cylinder undergoing controlled transverse oscillations at a Reynolds number of Re≈235. The dimensionless frequency and amplitude of cylinder oscillation were varied systematically over the ranges $0.2<f^{*}<1.8$ and $0.1<A^{*}<1.3$. A high-speed camera was used to capture the interference fringe patterns reflected from the soap film. These videos show the structure of the wake behind the cylinder, including the initial formation of vortices and the extended ‘vortex street’. Several wake patterns were identified, including the classic 2S, P+S, 2P, and 2T patterns, which are discussed in detail in the accompanying research article titled “The wake of a transversely oscillating circular cylinder in a flowing soap film at low Reynolds number” [1]. The videos presented in this article can be accessed through the Virginia Tech University Libraries’ Repository at https://doi.org/10.7294/14448027.v5[2].

## Specifications Table

**Table:** 

Subject	Mechanical Engineering
Specific subject area	Flow visualization from Vortex-Induced Vibrations (VIV) experiments in soap film
Type of data	Table, Video
How data were acquired	Camera: Falcon 4M60, Teledyne DALSA Lens: Carl Zeiss Distagon T* f/2.8-22 25 mm Acquisition software: Sapera CamExpert Data processing: MATLAB
Data format	Raw and analyzed
Parameters for data collection	Frame rate: 170 Hz. Exposure time: 500 *µ*s. Length: 1400 frames, 8.2 s
Description of data collection	A high-speed camera was used to record the interference fringe pattern reflected from an inclined soap film illuminated by a monochromatic light source.
Data source location	Institution: Virginia Polytechnic Institute & State University City/Town/Region: Blacksburg, Virginia 24060 Country: United States
Data accessibility	Repository name: Virginia Tech Data Repository Direct URL to data: https://doi.org/10.7294/14448027.v5 Instructions for accessing these data: 21 videos as .avi files can be downloaded individually or in one .zip file.
Related research article	W. Yang, E. Masroor, M.A. Stremler, The wake of a transversely oscillating circular cylinder in a flowing soap film at low Reynolds number, Journal of Fluids and Structures (2021) 105, 103343. [1]

## Value of the Data

• Researchers in the field of Vortex-Induced Vibrations (VIV) and Fluid-Structure Interac- tion (FSI), as well as fluid mechanicians interested in laminar flows and bluff body wakes, can benefit from these data.
• These flow-visualization videos reveal time-dependent flow structures in the wake of a transversely-oscillating circular cylinder in a gravity-driven soap film. Insight into the classic ‘2S’, ‘2P’, and ‘P+S’ type wakes, among others, can be obtained by examining the dynamic evolution of the flow and how it is affected by the cylinder's frequency ratio and oscillation amplitude.
• The ‘exotic wakes’ shown here are known to appear behind objects that are either free [3] or forced [4] to oscillate transverse to the direction of the background fluid flow. Con- trolled transverse oscillations of a circular cylinder, such as those used in this study, are a commonly-used technique to predict the nature and extent of the vortex-induced vibrations experienced by a cylindrical structure that is free to move in the transverse direction. The wake structures observed under controlled motion at a given frequency and amplitude are expected to be identical to those which arise from free vibrations at the same frequency and amplitude [5].
• Experimentalists can use these data as a benchmark when conducting similar experiments.
• These wake videos contain high resolution data of a two-dimensional, time-dependent system with significant spatio-temporal complexity that can be used as a platform for additional data analysis and technique development, including data-driven reconstruction of coherent structure dynamics, principal component analysis, neural network classification, and complex network analysis.

## 1 Data Description

**Fig. 1:**
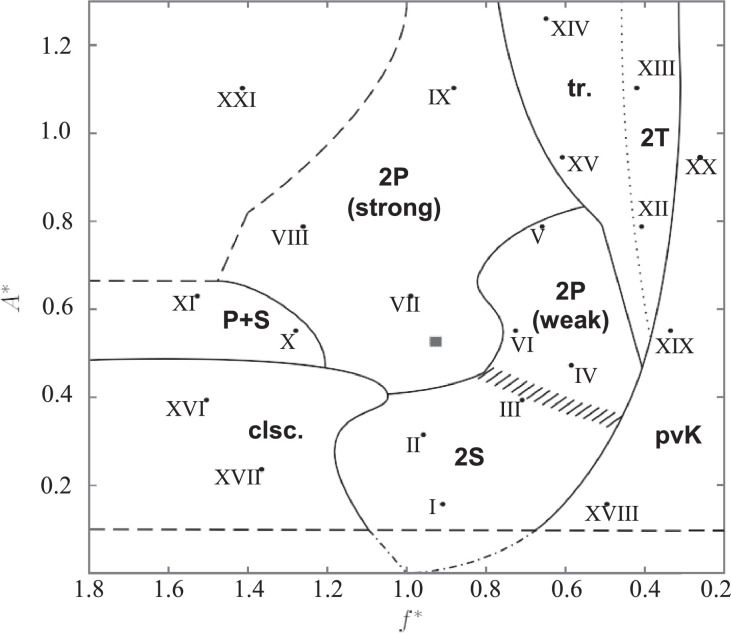
The frequency-amplitude parameter space covered by these experiments, adapted from [1]. The region labels and data points correspond to the classification of wake types summarized in Table 1. Each labeled point is associated with an experiment included in this article, providing examples of each wake type. For an explanation of each regime and the interpretation of the various lines, see [1].

This article includes video recordings of the flow past a circular cylinder that is forced to oscillate perpendicular to the mean background fluid velocity in a flowing soap film, providing a supplement to the static images in [1]. These videos show examples of different wake modes and their time-dependent evolution. Each of the 21 experiments included here correspond to a different set of oscillation frequency-amplitude pairs, which are summarized (in non-dimensional form) in Table 1 and plotted in the (*f* ^∗^*, A*^∗^)-plane in Fig. 1. The dimensionless frequency is given by $f^{*}\equiv f/f_{\text{St}}$, where *f* is the cylinder oscillation frequency and *f*_St_ is the natural frequency of vortex shedding when the cylinder is fixed. The dimensionless amplitude is given by $A^{*}\equiv A/D$, where *A* is the amplitude of the cylinder oscillation and *D* is the cylinder diameter.

**Table 1:** Summary of the experimental videos contained in this article. Roman numerals correspond to the data points shown in Fig. 1.

No.	*f*^∗^	*A*^∗^	Classification	file	Size (MB)
I	0.901	0.157	2S	1_Video_1mm_18p93.avi	23.33
II	0.951	0.315	2S	2_Video_2mm_17p93.avi	31.27
III	0.699	0.394	2S	3_Video_2p5mm_13p61.avi	28.7
IV	0.573	0.472	weak 2P	4_Video_3mm_11p29.avi	29.5
V	0.648	0.787	weak 2P	5_Video_5mm_12p7.avi	31.47
VI	0.715	0.551	weak 2P	6_Video_3p5mm_15p02.avi	28.91
VII	0.983	0.630	strong 2P	7_Video_4mm_18p59.avi	29.96
VIII	1.257	0.787	strong 2P	8_Video_5mm_24p65.avi	35.27
IX	0.873	1.102	strong 2P	9_Video_7mm_16p6.avi	34.81
X	1.277	0.551	P+S	10_Video_3p5mm_26p81.avi	32.69
XI	1.528	0.630	P+S	11_Video_4mm_28p890.avi	36.49
XII	0.394	0.787	2T	12_Video_5mm_7p72.avi	30.58
XIII	0.406	1.102	2T	13_Video_7mm_7p72.avi	30.65
XIV	0.638	1.260	transitional	14_Video_8mm_12p12.avi	27.64
XV	0.596	0.945	transitional	15_Video_6mm_11p790.avi	29.37
XVI	1.504	0.394	coalescing	16_Video_2p5mm_29p3.avi	28.85
XVII	1.364	0.236	coalescing	17_Video_1p5mm_25p73.avi	40.82
XVIII	0.482	0.157	perturbed von Kármán	18_Video_1mm_10p13.avi	22.65
XIX	0.320	0.551	perturbed von Kármán	19_Video_3p5mm_6p724.avi	26.95
XX	0.243	0.945	perturbed von Kármán	20_Video_6mm_4p814.avi	26.48
XXI	1.4147	1.102	uncharacterized	21_Video_7mm_26p89.avi	42.58

**Fig. 2:**
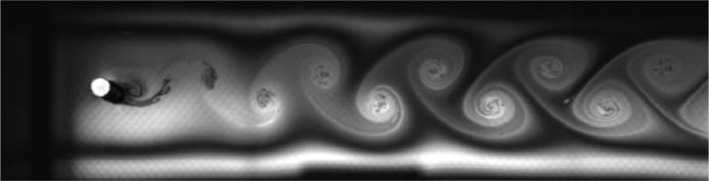
Exp. I: A 2S wake produced by a cylinder oscillating at *f*^∗^ = 0*.*901, *A*^∗^ = 0*.*157. The vortices are connected to each other in an unbroken braid shear layer and, once developed into a vortex street, translate downstream without significant change of shape or relative position. (1_Video_1mm_18p93.avi)

**Fig. 3:**
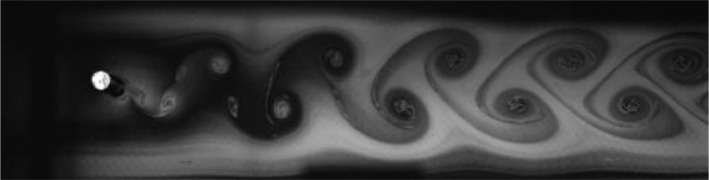
Exp. II: A 2S wake produced by a cylinder oscillating at *f*^∗^ = 0*.*951, *A*^∗^ = 0*.*315. This wake most clearly resembles the 2S mode in the mid-to-far wake. During the first half of the video, the ‘stray vorticity’ in the braid shear layer connecting two successive vortices is relatively strong in the near-to-mid wake region, but this vorticity does not persist into the far wake. In the second half of the video, this stray vorticity has significantly weakened and the wake appears purely 2S. (2_Video_2mm_17p93.avi)

**Fig. 4:**
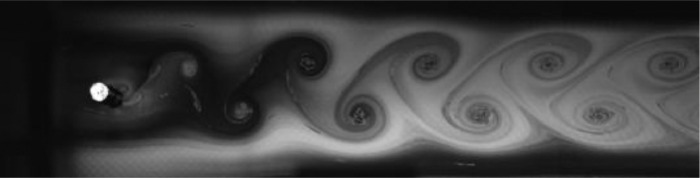
Exp. III: A 2S wake produced by a cylinder oscillating at *f*^∗^ = 0*.*699, *A*^∗^ = 0*.*394. For the duration of the experiment the wake behavior here is similar to that in the first half of Exp. II (Fig. 3). Thus, this wake may be considered ‘weak 2P’ for the first few diameters downstream and 2S in the mid-to-far wake. This wake, and comparison with Exp. II, demonstrates the subjectivity of identifying the boundary between the 2S and 2P regions. (3_Video_2p5mm_13p61.avi)

**Fig. 5:**
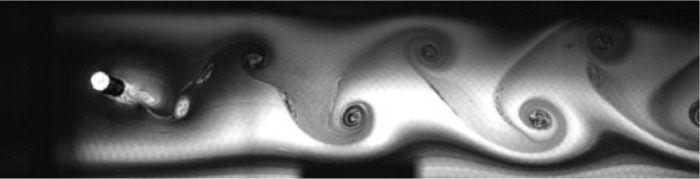
Exp. IV: A weak 2P wake produced by a cylinder oscillating at *f*^∗^ = 0*.*573, *A*^∗^ = 0*.*472. During each oscillation cycle, a clockwise vortex from the top is followed — after a noticeable gap — by a small counterclockwise vortex, which is then followed immediately by a large counterclockwise vortex, then another gap and a small clockwise vortex. Thus, this weak 2P wake takes on a horizontally inhomogeneous appearance with large regions of irrotational fluid amongst the vortices. (4_Video_3mm_11p29.avi)

**Fig. 6:**
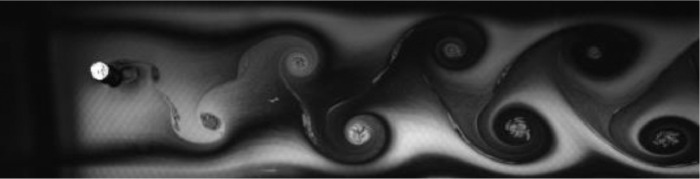
Exp. V: A weak 2P wake produced by a cylinder oscillating at *f*^∗^ = 0*.*648, *A*^∗^ = 0*.*787. Two pairs of vortices are shed in each cycle, but the weaker member of each pair weakens considerably in the far wake. This weak vortex continues to remain visible in the far wake as an elongated region of vorticity. (5_Video_5mm_12p7.avi)

**Fig. 7:**
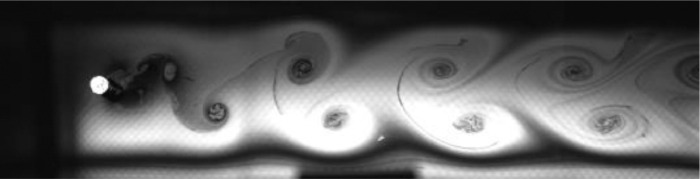
Exp. VI: A cylinder oscillating at *f*^∗^ = 0*.*715, *A*^∗^ = 0*.*551 produces a weak 2P wake. The weaker member of each pair clearly persists into the far wake. (6_Video_3p5mm_15p02.avi)

**Fig. 8:**
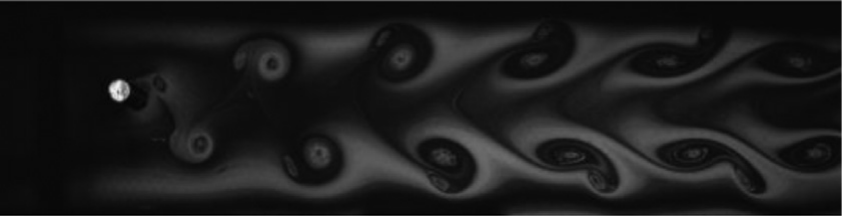
Exp. VII: A strong 2P wake produced by a cylinder oscillating at *f*^∗^ = 0*.*983, *A*^∗^ = 0*.*630. The vortices in each pair are shed close together, giving rise to a strongly paired structure that clearly persists into the far wake. The weaker member of the pair orbits the stronger member while each pair structure moves roughly parallel to the wake centerline. (7_Video_4mm_18p59.avi)

**Fig. 9:**
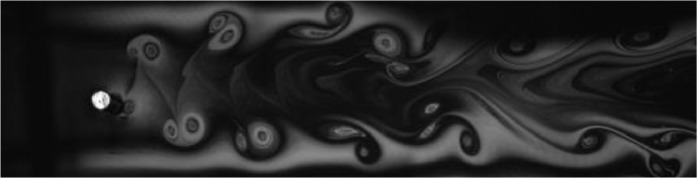
Exp. VIII: A strong 2P wake produced by a cylinder oscillating at *f*^∗^ = 1*.*257, *A*^∗^ = 0*.*787. Near-to-mid wake behavior is similar to that in Exp. VII (Fig. 8), but in the far wake region there is significant inter-vortex motion, with some pairs displaying large transverse motion leading to intermittent break-up of the wake structure, e.g., at 00:06. (8_Video_5mm_24p65.avi)

**Fig. 10:**
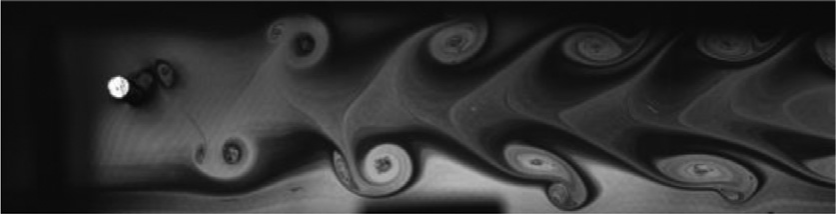
Exp. IX: A strong 2P wake produced by a cylinder oscillating at *f*^∗^ = 0*.*873, *A*^∗^ = 1*.*102. Overall behavior is similar to that of Exp. VII (Fig. 8), but in this case there is greater lateral spacing, which occasionally makes it difficult to identify the top vortex pair. In contrast to Exp. VIII (Fig. 9), this strong 2P wake is ‘well-behaved’. (9_Video_7mm_16p6.avi)

**Fig. 11:**
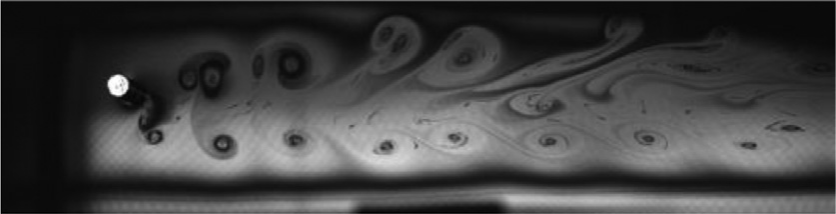
Exp. X: A P+S wake produced by a cylinder oscillating at *f*^∗^ = 1*.*277, *A*^∗^ = 0*.*551. A pair of vortices is shed from the top of the cylinder and a single vortex from the bottom. Occasionally, an extra vortex is shed or lateral vortex motion occurs in the far wake, but the overall structure is that of the asymmetric P+S wake. (10_Video_3p5mm_26p81.avi)

**Fig. 12:**
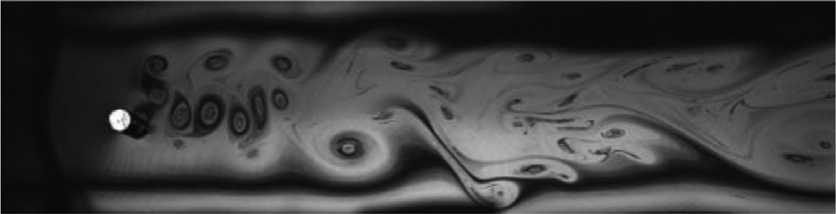
Exp. XI: A P+S wake produced by a cylinder oscillating at *f*^∗^ = 1*.*528, *A*^∗^ = 0*.*630. A pair of vortices is shed from the bottom side of the cylinder and a single vortex from the top. For these parameters the wake structure is quite unstable and irregular, frequently becoming incoherent in the mid-to-far wake region. (11_Video_4mm_28p890.avi)

**Fig. 13:**
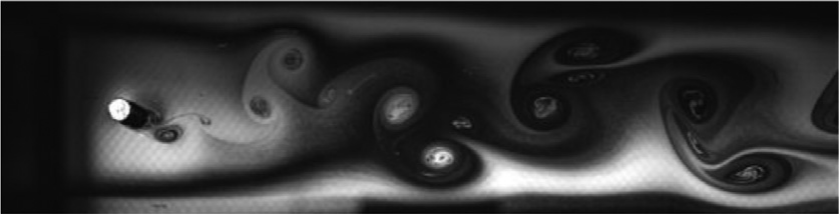
Exp. XII: A 2T wake is produced by a cylinder oscillating at *f*^∗^ = 0*.*394, *A*^∗^ = 0*.*787. Each triplet consists of two strong vortices that convect downstream with little change of shape or relative motion and one weak vortex that moves between the strong vortices. (12_Video_5mm_7p72.avi)

**Fig. 14:**
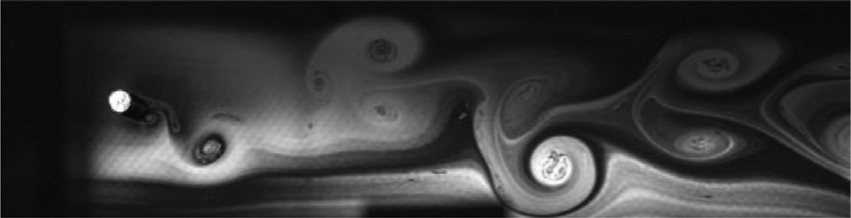
Exp. XIII: A 2T wake produced by a cylinder oscillating at *f*^∗^ = 0*.*406, *A*^∗^ = 1*.*102. Two triplets of vortices are shed in each oscillation cycle. (13_Video_7mm_7p72.avi)

**Fig. 15:**
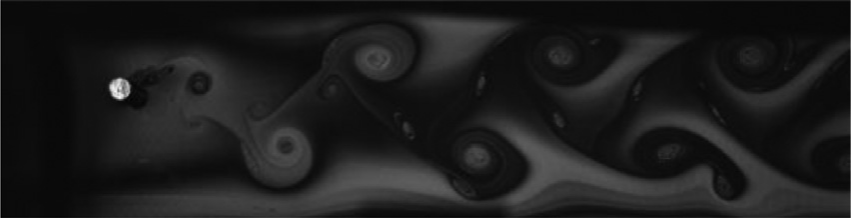
Exp. XIV: A ‘transitional’ wake produced by a cylinder oscillating at *f*^∗^ = 0*.*638, *A*^∗^ = 1*.*260. This type of transitional wake exhibits a synchronized structure, in which multiple vortices of varying sizes are shed during each oscillation cycle. These vortices convect downstream with little relative motion. (14_Video_8mm_12p12.avi)

**Fig. 16:**
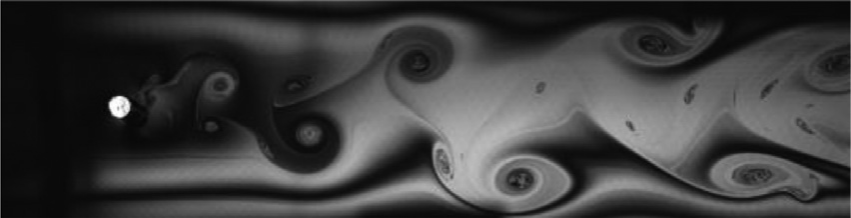
Exp. XV: A ‘transitional’ wake produced by a cylinder oscillating at *f*^∗^ = 0*.*596, *A*^∗^ = 0*.*945. Although vortex shedding is synchronized with the cylinder's motion, no discernible patterns are established in the wake, which is much more disorganized than in Exp. XIV, Fig. 15. (15_Video_6mm_11p790.avi)

**Fig. 17:**
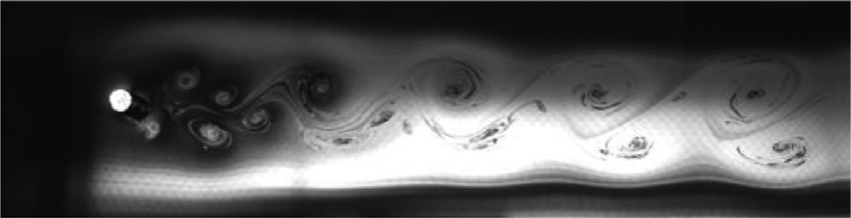
Exp. XVI: A coalescing wake produced by a cylinder oscillating at *f*^∗^ = 1*.*504, *A*^∗^ = 0*.*394. Several vortices are shed in each cycle, crowding the near wake until approximately 5 diameters downstream, where they coalesce into a large-scale 2S wake comprised of the amalgamated vortices. (16_Video_2p5mm_29p3.avi)

**Fig. 18:**
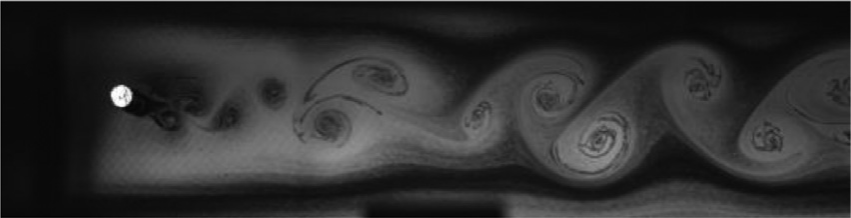
Exp. XVII: A coalescing wake produced by a cylinder oscillating at *f*^∗^ = 1*.*364, *A*^∗^ = 0*.*236. A large number of small vortices are shed during each oscillation cycle, and these rapidly merge into larger vortex structures that become arranged roughly in a 2S wake. (17_Video_1p5mm_25p73.avi)

**Fig. 19:**
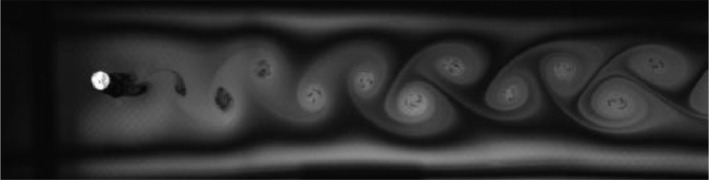
Exp. XVIII: A perturbed von Kármán wake produced by a cylinder oscillating at *f*^∗^ = 0*.*482, *A*^∗^ = 0*.*157. Because of the low oscillation amplitude, this wake looks very similar to a synchronized 2S wake; however, the vortex shedding is at the Strouhal frequency, not synchronized with the cylinder oscillation. (18_Video_1mm_10p13.avi)

These experiments provide representative samples of each of the 8 wake types, or modes, identified and discussed in [1]:

• **2S**, for which two vortices of opposite sign are generated in each shedding cycle, synchronized with the oscillatory motion of the cylinder;
• **weak 2P**, for which two pairs of vortices with glide-reflective symmetry are shed in each cycle, but the weaker member of each pair may not persist in the far wake, which can there resemble a 2S wake;
• **strong 2P**, for which two pairs of vortices with glide-reflective symmetry are shed in each cycle, and these pairs persist in the far wake;
• **P**+**S**, an asymmetric mode for which one pair of vortices and then a single vortex are generated from opposite sides of the cylinder during each shedding cycle;
• **2T**, for which two triplets of vortices with glide-reflective symmetry are shed in each oscillation cycle;
• **transitional**, a mode that corresponds with the generation of a non-canonical pattern or with no consistent pattern being sustained for any significant length of time during any experiment;
• **coalescing**, with a large number of vortices generated in the near wake that coalesce into a 2S-like mode in the mid-to-far wake; and
• **perturbed von Kármán**, in which vortex shedding continues to occur at the natural frequency and does not synchronize with the cylinder's motion, producing a wake structure that be interpreted as a 2S wake superimposed along a sinusoidal ‘centerline’ caused by the cylinder's motion.

A detailed description of the wake dynamics exhibited by each of these modes is included in [1]. We also include one example of an experiment (XXI) from the ‘uncharacterized’ region in the top-left part of the wake mode map.

**Fig. 20:**
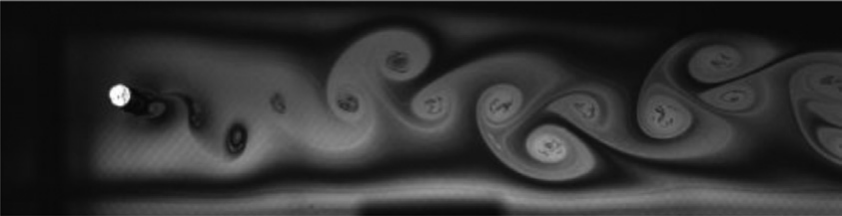
Exp. XIX: A perturbed von Kármán wake produced by a cylinder oscillating at *f*^∗^ = 0*.*320, *A*^∗^ = 0*.*551. Vortices are shed at the Strouhal frequency along a sinusoidal path that has a substantially larger amplitude than in Exp. XVIII (Fig. 19). The resulting arrangement of the vortices in the far wake gives the appearance of a 2T-like structure. (19_Video_3p5mm_6p724.avi)

**Fig. 21:**
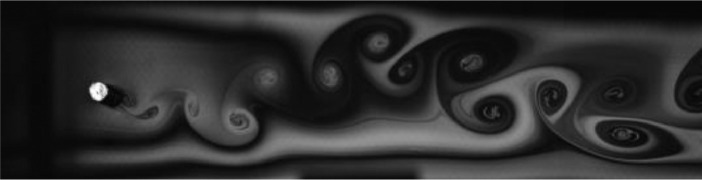
Exp. XX: A perturbed von Kármán wake produced by a cylinder oscillating at *f*^∗^ = 0*.*243, *A*^∗^ = 0*.*945. The large-amplitude, long-wavelength sine curve traced by the cylinder relative to the background flow is even more apparent than in Exp. XIX, Fig. 20. (20_Video_6mm_4p814.avi)

**Fig. 22:**
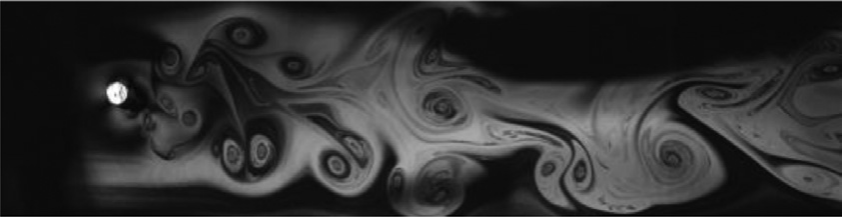
Exp. XXI: A wake from the ‘uncharacterized’ region produced by a cylinder oscillating at *f*^∗^ = 1*.*4147, *A*^∗^ = 1*.*102. Out-of-plane motion of the soap film causes dark spots to appear in the interferograms, highlighting the intermittent deformation of the film throughout the field of view. (21_Video_7mm_26p89.avi)

## 2 Experimental Design, Materials and Methods

An inclined, gravity-driven soap film channel similar to that described by [6], [7], [8] was used to generate the data presented in this article.

### 2.1 The soap film system

**Fig. 23:**
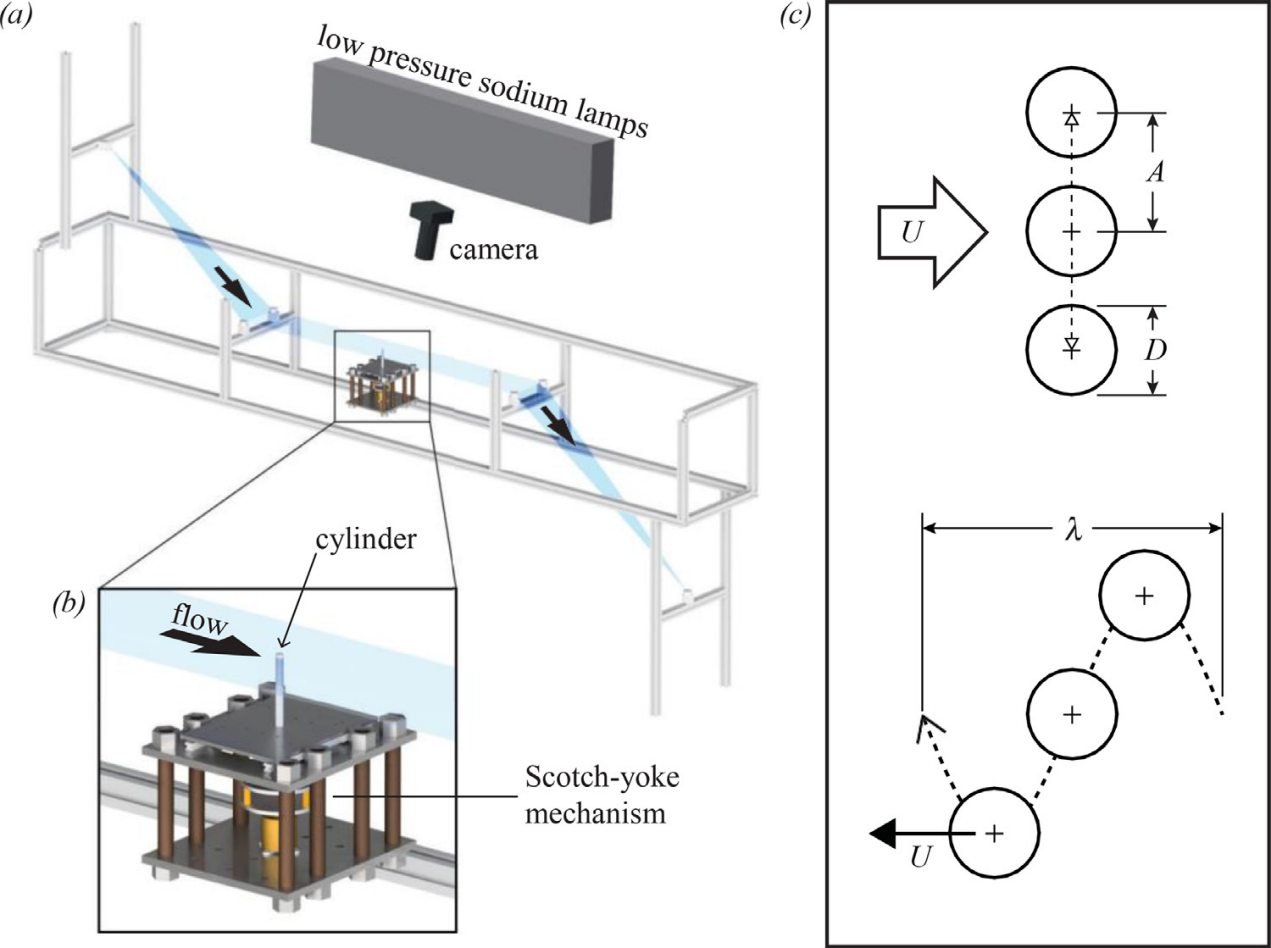
(a) Schematic of the experimental system and (b) detailed view of the cylinder oscillation mechanism, from [1]. A photo of the experimental system and a video of the oscillating cylinder mechanism in operation are included in the data repository [2]. (c) Illustration of the cylinder motion (top) transverse to a background flow or (bottom) along a sinusoidal path through a quiescent fluid.

The experimental apparatus was built around an aluminum frame, shown schematically in Fig. 23(a). At the top of the frame, a reservoir (not shown) held fresh soap solution, which flowed through a valve into the apex of two nylon wires attached to the frame. These nylon wires were held taut to form three distinct stages: an expanding stage, in which the wires descended steeply at an angle of 57° with respect to the horizontal and expanded to a width of approximately 10 cm; the main test section, in which they descended at an inclination angle of 14° while aligned in parallel approximately 10 cm apart for 1 m; and finally a contracting stage, in which they descended at an angle of 68° and converged to the bottom of the frame where they met in an apex. The inclination angle of 14° in the test section was selected as a compromise between keeping the mean film speed low while preventing excessive sagging of the soap film in the test section.

A constant pressure head was maintained at the top of the soap film by using two nested containers of soap solution, with the outer container serving as an ‘overflow reservoir’ from which solution was continually pumped into the (brimming) inner container. This soap solution source ensured that the driving pressure did not vary over the course of an experiment through depletion of the solution. In all experiments, the solution was 1% by volume of Dawn *Escapes^TM^* liquid soap (Proctor & Gamble) in water.

After the upper valve was opened, soap solution flowed onto the wires and a ‘bubble’ film comprising this solution was manually drawn over the entire length of the channel between the nylon guide wires using a bar of acrylic. Once the soap ‘bubble’ was successfully extended between the nylon wires, soap solution began to flow from the reservoir at the top of the system to a collection bucket at the bottom (not shown). In Fig. 23(a), the developed soap film bounded on both sides by nylon guide wires is shown schematically in translucent blue.

### 2.2 Cylinder oscillation

A circular cylinder with diameter *D* = 6*.*35 mm (= 0*.*25 in) was mounted perpendicular to the plane of the experimental section of the soap film as shown in Fig. 23(b) and oscillated transverse to the flow direction. As shown schematically in Fig. 23(c), the cylinder was made to oscillate with amplitude *A* and frequency *f* (or wavelength *λ* when viewed in a frame of reference moving with the fluid). This oscillation was accomplished by securely attaching the cylinder to a scotch-yoke mechanism that was driven steadily using a motor mounted on a vibration table. The rotational motion of the motor was transformed by the scotch-yoke mechanism into a sinusoidally-varying, linear transverse motion.

Two non-dimensional numbers govern the cylinder's oscillation: the reduced amplitude $A^{*}\equiv A/D$, and either a non-dimensional wavelength $\lambda^{*}\equiv \lambda /D$ or a non-dimensional frequency $f^{*}\equiv f/f_{\text{St}}$. Here, *λ* is the wavelength of the cylinder's motion in a frame of reference moving with the free stream, and *f*_St_ is the natural frequency of vortex shedding from the cylinder when it is held stationary, often called the *Strouhal frequency*. In some studies (see, e.g., Ref. [4]), results are presented using the (equivalent) reduced velocity, $U^{*}\equiv U/\left(fD\right)=\lambda /D$, where *U* is the (steady) speed of the body relative to the flow in the streamwise direction. The dimensionless frequency and reduced velocity are related through the Strouhal number,

(1) $$\text{St}\equiv \frac{f_{\text{St}}D}{U},$$

as^1^ St f*U* = 1. For $10^{3}\leq \text{Re}\leq 10^{5}$, the Strouhal number is approximately constant for a smooth circular cylinder, and *U*^∗^ and 1*/ f* ^∗^ can be used interchangeably. However, for the data reported here, the relationship between *f* ^∗^ and *U*^∗^ is Reynolds number dependent.

**Fig. 24:**
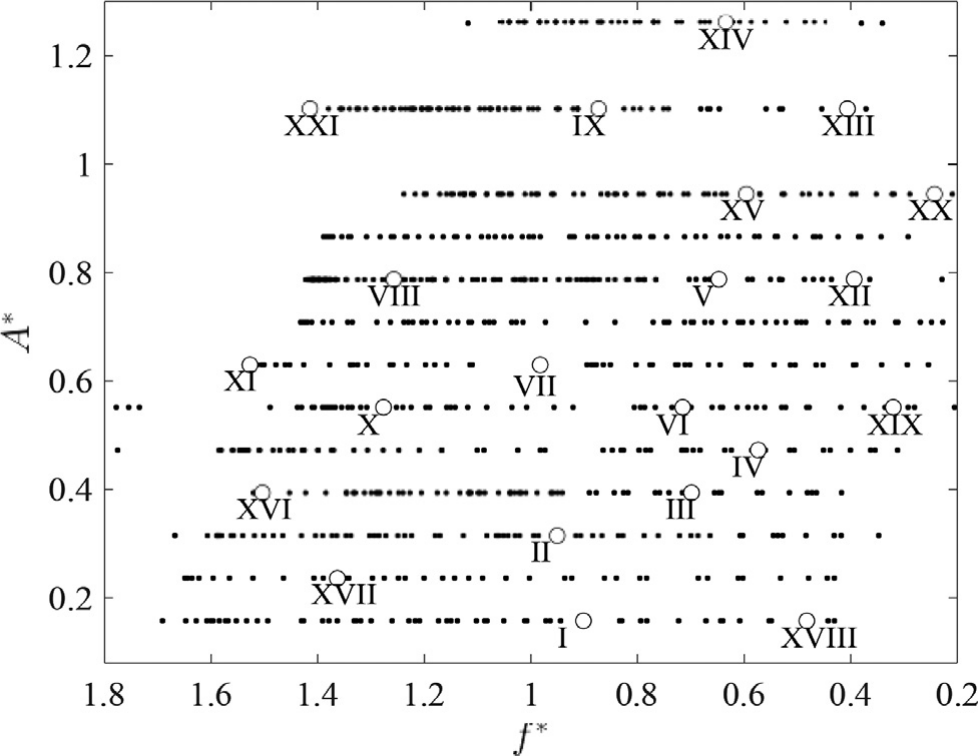
Nondimensionalized frequency and amplitude of the data points collected for this work. Small filled circles represent data points that were analyzed in order to generate Fig. 1, and large open circles mark the locations of the 21 data points included in this article, labeled with the corresponding Roman numerals.

In this work, we used *f* ^∗^ and *A*^∗^ to characterize the cylinder's oscillation, yielding the two-dimensional ‘parameter space’ map of wake patterns shown in Fig. 1. The range of variables *f* ^∗^ and *A*^∗^ for which data were collected is shown in Fig. 24, with each video represented by a point in the (*f* ^∗^*, A*^∗^) plane. Data were collected intermittently in the frequency range $0.2<f^{*}<1.8$ and for thirteen amplitude values in the range $0.1<A^{*}<1.3$; our collected data points are shown in Fig. 24. The *f* ^∗^ and *A*^∗^ values corresponding to the experiments included here are provided in Table 1.

### 2.3 Illumination and flow visualization

The soap film was illuminated with a three-phase monochromatic sodium lamp assembly, as shown schematically in Fig. 23(a). A high-speed camera was placed near the light source and positioned so that it received the light from the sodium lamp as reflected from the soap film. The videos recorded by the camera capture the interference pattern from light reflected from the upper and lower surfaces of the soap film; very small variations in the thickness result in changes in the interference pattern [9], leading to the flow visualization documented in these videos. Thus, flowing soap films provide us with a rich ‘two-dimensional laboratory’ for hydrodynamics experiments with minimally-invasive, particle-free flow visualization.

Videos of the wake were recorded at a frame rate of 170 Hz with a window of 2352×600 pixels for 1400 frames or approximately 8.2 seconds. The resulting videos were then compressed using MATLAB's Video Processing Toolbox. A total of 839 videos were recorded in order to produce the wake-mode map and conduct the analysis in [1]; only a representative sample of each type of wake is included here, as illustrated in Fig. 24.

Municipal electricity in the United States is supplied at 60 Hz AC. Because our frame rate was higher than this at 170 Hz, we supplied power to the sodium lamp using three independent phases of AC that were shifted 120° relative to each other. This ensured that the soap film was illuminated approximately evenly in time and prevented a peak in the power spectrum at 60 Hz that would have corrupted the data.

### 2.4 Vortex identification

The illumination and visualization of soap films using interferometry, as was done in this study, is a standard technique in the field, going back at least to 1984 with the work of Couder et al. [10]. The scalar field that is captured in the video images in this study is related to the small changes in thickness that occur in a soap film due to the Marangoni effect, in which the thickness plays the role of density and the surfactant concentration plays the role of pressure in an incompressible Navier-Stokes fluid [11]. It has long been recognized by experimentalists that interferometry results are closely related to the hydrodynamic flow field, and a number of important fluid dynamics results have been replicated in soap films; see, e.g., Refs. [12,13]. In addition, Gillissen *et. al* [14] have developed a technique to reconstruct the velocity field from the scalar field obtained using interferometry of a soap film.

Recently, Eshraghi and collaborators [15] investigated the wake of a stationary circular cylinder in a flowing soap film using multiple interrogation techniques. They showed that the identification of vortex centers as circular regions in the interferometry images accurately predicts the true location of vortex centers as determined using the velocity fields obtained with digital particle image velocimetry (DPIV). In this work, we manually identified circular or nearly-circular structures, usually accompanied by rapid changes in image intensity, as vortices in the cylinder wake.

### 2.5 Soap film characteristics

Although the wake patterns display significant spatio-temporal complexity, these flows remained laminar. The design of the soap film channel — including the length, width and angle of inclination of the three sections — was chosen in order to achieve an approximately constant velocity throughout the test section, following Georgiev and Vorobieff [6]. Using particle-tracking velocimetry, we determined this mean background velocity to be *U* = 680 mm/s in our experiments.

Due to the difficulty of measuring the viscosity of a soap film, it is common to use an indirect approach to determine the Reynolds number of a flowing soap film. Gharib and Derango [16] proposed using the Strouhal number (1) for flow past an isolated cylinder to estimate the Reynolds number in a soap film, and Wen and Lin [17] showed that the Strouhal–Reynolds number relationship for a cylinder in a soap film is in good agreement with established results [18]. We used this approach to determine the Reynolds number in our experiments by measuring the vortex-shedding frequency *f*_St_ and flow speed *U* for the flow past a stationary cylinder before each set of experiments. Using the St–Re relationship from Ref. [19],

(2) $$\text{St}=0.2731-1.1129\;Re^{-1/2}+0.4821\;Re^{-1},$$

each St value was then converted into a Reynolds number. The Reynolds number for these experiments was determined to be Re=235±14.

Elastic effects in the film were determined to be negligible. By observing the change of solution level in the overflow reservoir, we measured the volumetric flowrate during steady-state operation, and using mass conservation together with an assumption of uniform flow we estimated the average film thickness to be h≈3μm. The elastic Mach number, $M_{e}\equiv U/U_{M}$, characterizes the significance of compressible-like effects in the film, where $U_{M}\equiv {\left[2E_{M}/\left(\rho h\right)\right]}^{1/2}\;$is the Marangoni wave speed. Assuming the Marangoni elasticity is $E_{M}\approx 22\;\text{mN}/m$ [20] and the density is $\rho \approx 1g/cm^{3}$, the elastic Mach number for these experiments was $M_{e}\approx 0.2$. Thus, the flow of this film can be considered as an acceptable approximation of a two-dimensional, incompressible Newtonian fluid [11].

The interferometry images are made possible by thickness variations in the film, but these thickness variations were judged to not interfere with the fluid dynamics. In the absence of the cylinder, the film was approximately a uniform thickness throughout the test section, consistent with the observations of Refs. [21] and [6]. For flow past a circular cylinder, Ref. [15] shows that the resulting flow structures in the wake produce thickness variations that are less than 25% of the average film thickness for 200≤Re≤300. By the analogy between thickness of the film and density of a Navier–Stokes fluid, these thickness variations produce a small Boussinesq-like buoyancy effect that can be considered negligible [11].

**Fig. 25:**
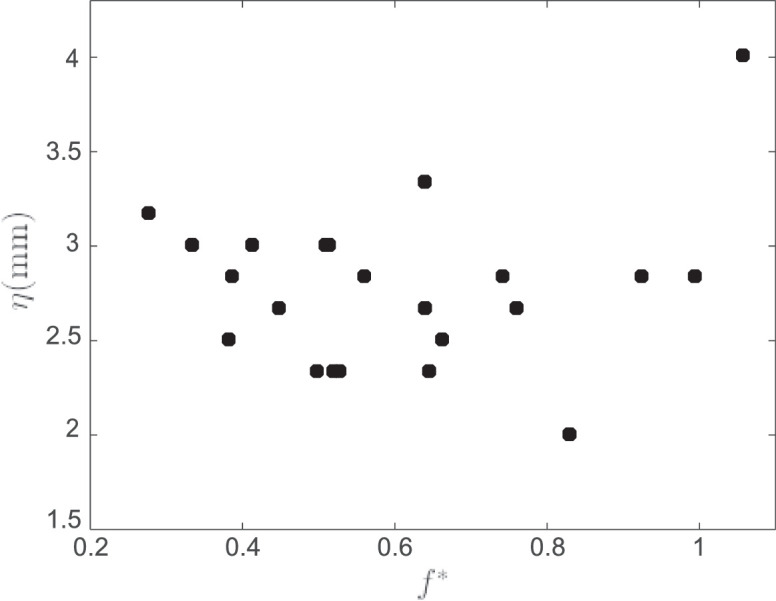
Maximum observed out-of-plane displacement *η* of the soap film against dimensionless oscillation frequency f* for an oscillation amplitude of *A* = 6 mm, corresponding to *A*^∗^ = 0*.*94.

The wake patterns in the regimes of interest, as represented by examples I–XX, can be approximated as occurring in a planar film. In the undisturbed film, gravity caused an observed out-of-plane deformation of at most η≤0.5mm, or a relative displacement of η/W≤0.5%, where *W* = 10 cm is the width of the test section. Oscillation of the cylinder caused additional out-of-plane displacement of the film. For the large values of oscillation amplitude and frequency achieved in the ‘uncharacterized’ region of Fig. 1, the out-of-plane displacement of the film was large enough to cause dark areas to appear in the interferometry images. As seen in the video accompanying Fig. 22, these regions of relatively large out-of-plane displacement appear intermittently throughout the film, including near the cylinder. To quantify the amplitude of these film displacements, we placed a video camera near the end of the film and oriented it to point directly upstream, creating an end-on view of the film. We monitored the out-of-plane displacement *η* of the soap film with the cylinder undergoing oscillations at a relatively high amplitude of *A*^∗^ = 0*.*94 (*A* = 6 mm) for several frequencies in the range $0.2<f^{*}<1.1$, where $f^{*}\approx 1$ corresponds to the edge of the ‘uncharacterized’ region at this oscillation amplitude. As shown in Fig. 25, we observed a maximum peak-to-peak out-of-plane displacement amplitude of η≈4mm in the neighborhood of the cylinder. We extrapolate from this observation an estimate that the out-of-plane displacement in this system was η≤5mm for all but the ‘uncharacterized’ region, corresponding to a relative displacement η/W≤5%. Out-of-plane displacement of the soap film was thus assumed to have a negligible effect on the wake dynamics in examples I–XX.

## CRediT Author Statement

**Emad Masroor:** Formal analysis, Software, Data curation, Validation, Visualization, Writing – original draft, review & editing; **Wenchao Yang:** Conceptualization, Methodology, Investigation, Software, Formal analysis, Writing – review & editing; **Mark A. Stremler:** Conceptualization, Supervision, Formal analysis, Writing – review & editing.

## Declaration of Competing Interest

The authors declare that they have no known competing financial interests or personal relationships which have, or could be perceived to have, influenced the work reported in this article.

## Footnotes

1 This equation corrects an error in Ref. [1].
